# Matching Patients to Clinical Trials with Large Language Models

**Published:** 2024-04-20

**Authors:** Qiao Jin, Zifeng Wang, Charalampos S. Floudas, Fangyuan Chen, Changlin Gong, Dara Bracken-Clarke, Elisabetta Xue, Yifan Yang, Jimeng Sun, Zhiyong Lu

**Affiliations:** 1. National Center for Biotechnology Information (NCBI), National Library of Medicine (NLM), National Institutes of Health (NIH).; 2. Department of Computer Science, University of Illinois Urbana-Champaign.; 3. Center for Immuno-Oncology, Center for Cancer Research, National Cancer Institute, National Institutes of Health.; 4. School of Medicine, University of Pittsburgh.; 5. Jacob Medical Center, Albert Einstein College of Medicine.; 6. School of Computer Science, University of Maryland College Park.

## Abstract

Clinical trials are often hindered by the challenge of patient recruitment. In this work, we introduce TrialGPT, a first-of-its-kind large language model (LLM) framework to assist patient-to-trial matching. Given a patient note, TrialGPT predicts the patient’s eligibility on a criterion-by-criterion basis and then consolidates these predictions to assess the patient’s eligibility for the target trial. We evaluate the trial-level prediction performance of TrialGPT on three publicly available cohorts of 184 patients with over 18,000 trial annotations. We also engaged three physicians to label over 1,000 patient-criterion pairs to assess its criterion-level prediction accuracy. Experimental results show that TrialGPT achieves a criterion-level accuracy of 87.3% with faithful explanations, close to the expert performance (88.7%–90.0%). The aggregated TrialGPT scores are highly correlated with human eligibility judgments, and they outperform the best-competing models by 32.6% to 57.2% in ranking and excluding clinical trials. Furthermore, our user study reveals that TrialGPT can significantly reduce the screening time (by 42.6%) in a real-life clinical trial matching task. These results and analyses have demonstrated promising opportunities for clinical trial matching with LLMs such as TrialGPT.

## Introduction

Clinical trials examine the effectiveness of medical interventions and provide crucial evidence that can be used to guide clinical practice. They also offer an opportunity for participants to receive experimental treatments that could potentially improve their health outcomes. However, matching patients to suitable clinical trials can be a challenging process^1–3^. This process includes analyzing a patient’s medical history, understanding the eligibility criteria of each clinical trial, and ensuring a match that satisfies both patient needs and trial requirements. As such, manually matching patients and clinical trials is often labor-intensive, time-consuming, and prone to human errors.

Recently, artificial intelligence (AI) has shown promise in improving the efficiency and accuracy of patient-trial matching^4^. Based on the directionality, there are two types of patient-trial matching tasks. The “trial-to-patient” scheme matches one trial to a list of candidate patients, which is a common need for clinical trial organizers and can be done by converting the trial criteria to structured query languages and searching the patient database^5–7^. On the other hand, the “patient-to-trial” scheme matches one patient to a list of candidate clinical trials^8–11^. In this study, we focus on the patient-centric “patient-to-trial” scheme because such a model can empower individual patients as well as referral offices to explore a large set of potentially eligible clinical trials. However, the heterogeneity and ambiguity inherent in patient records and clinical trial criteria induce significant challenges for AI algorithms. Prior efforts encoded patient records and trial criteria into dense embeddings using neural networks, aiming to represent them in the same embedding space that enables patient-trial matching through similarity search^12–14^. Nonetheless, training neural networks with the language understanding capability of ambiguous criteria texts and diverse patient records requires large datasets. This is often infeasible due to the lack of paired patient-criterion matching annotations. Besides, the black-box dense retrieval process is not explainable, and thus hard to debug when extrapolating it to previously unseen criteria and patients.

In this work, we aim to evaluate how recent large language models (LLMs) such as GPT-4^15^ can aid the process of patient-to-trial matching in a data-efficient and transparent way. LLMs are transformer-based models^16^ that can understand a given context and generate human-like responses accordingly. They have shown state-of-the-art capabilities in both the general domain^15,17^ and biomedicine^18^, including question answering^19–24^ and clinical trial design^25,26^. Several pilot studies have also explored using LLMs to enhance the first-stage retrieval of clinical trials through information extraction^27^, perform data augmentation with clinical trial criteria^28^, and structuralize clinical trial criteria^29^, while our study pivots on rationalizing the criteria-level predictions and the fine-grained patient-to-trial ranking with LLMs.

**Figure 1.**
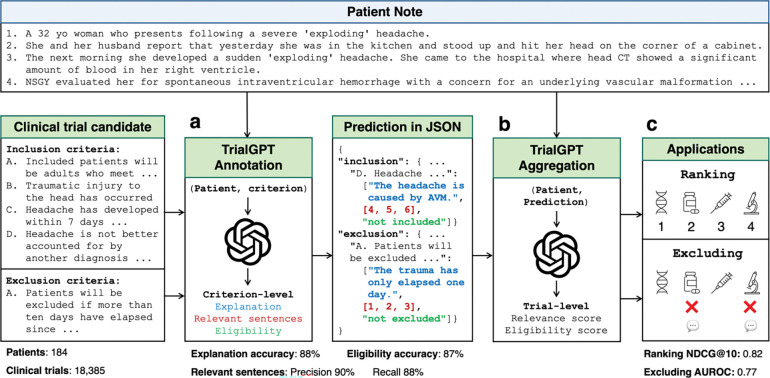
The overall architecture of TrialGPT. **(a)** Given a patient note and a clinical trial candidate as input, TrialGPT first uses LLMs to generate the relevance explanation, relevant sentence locations, and the patient eligibility for each criterion. **(b)** TrialGPT then uses LLMs to aggregate criterion-level predictions into trial-level scores. **(c)** The trial-level scores can be used to rank or exclude a list of clinical trial candidates for the given patient.

We propose TrialGPT, a novel LLM framework for patient-to-trial matching. As shown in Figure 1, TrialGPT handles two challenging tasks: (a) predicting patient eligibility for each clinical trial criterion with explanations, (b) aggregating the criterion-level predictions to generate a trial-level score for downstream applications. Specifically, given a patient note and a candidate clinical trial, TrialGPT predicts three elements for each eligibility criterion: (1) a natural language explanation showing the relevance of the patient to the criterion, (2) locations of relevant sentences in the patient note that are relevant to the target criterion, and (3) the eligibility classification indicating whether the patient meets this criterion. Three domain experts evaluate TrialGPT on 1,015 patient-criterion pairs, and the results show that TrialGPT can accurately explain patient-criterion relevance, locate relevant sentences, and predict criterion-level eligibility with an accuracy close to that of human experts. We then evaluate the trial-level scores by TrialGPT on three publicly available cohorts of 184 patients and 18,238 manually annotated clinical trials. Experimental results show that the aggregated-TrialGPT scores are highly correlated with expert eligibility annotations. They can be used to match eligible trials with patients effectively and exclude ineligible trials, with a performance of 11.3% to 27.4% higher than the best baselines.

We also conducted a pilot user study that mimics the actual clinical trial matching task at the National Cancer Institute (NCI). In the evaluation, each patient-trial pair is evaluated by one medical expert with TrialGPT and another one without TrialGPT. We also ensure that each medical expert annotates half of the pairs with TrialGPT and half without to mitigate the skill differences between the annotators when computing the time reduction. The overall time saving for all patient-trial pairs is about 42.6% (from 61.5 seconds to 31.3 seconds), which shows the potential to greatly enhance the efficiency of the clinical trial matching process.

## Results

### Cohort characteristics

To evaluate TrialGPT, we use the patient notes and clinical trials from three publicly available cohorts: a test collection for patient-trial matching published by Special Interest Group on Information Retrieval (SIGIR) in 2016^9^, and the 2021 and 2022 Clinical Trials (CT) tracks^8^ of the Text REtrieval Conference (TREC). For each patient, we sample at most 50 clinical trials for each eligibility category: the TREC CT cohorts have “eligible”, “excluded” (“ineligible”) and “irrelevant” trials, while the SIGIR cohort does not have the “excluded” trials. The baseline statistics of these patient cohorts are shown in Table 1. We use the combination of these three cohorts as the final evaluation corpus.

For evaluating criterion-level prediction accuracy, three physicians were recruited and manually annotated 1,015 patient-criterion pairs regarding three output elements as shown in Figure 1a: (1) the correctness of TrialGPT relevance explanation between the given patient and the criterion, (2) the relevant sentence locations in the patient note, and (3) the criterion-level prediction of the given patient’s eligibility. Consensus annotations derived from individual annotations and further discussions are used as the ground truth.

### TrialGPT achieves a high criterion-level prediction accuracy

As shown in Figure 1a, TrialGPT first generates the rationales and the relevant sentences for each criterion. Then, it predicts the criterion-level eligibility classification based on the rationales. TrialGPT assigns each inclusion criterion a label within {Included, Not included, Not enough information, Not applicable} and each exclusion criterion a label within {Excluded, Not excluded, Not enough information, Not applicable}. The label definitions can be found in the Online Methods.

### Evaluating relevance explanations

**Figure 2.**
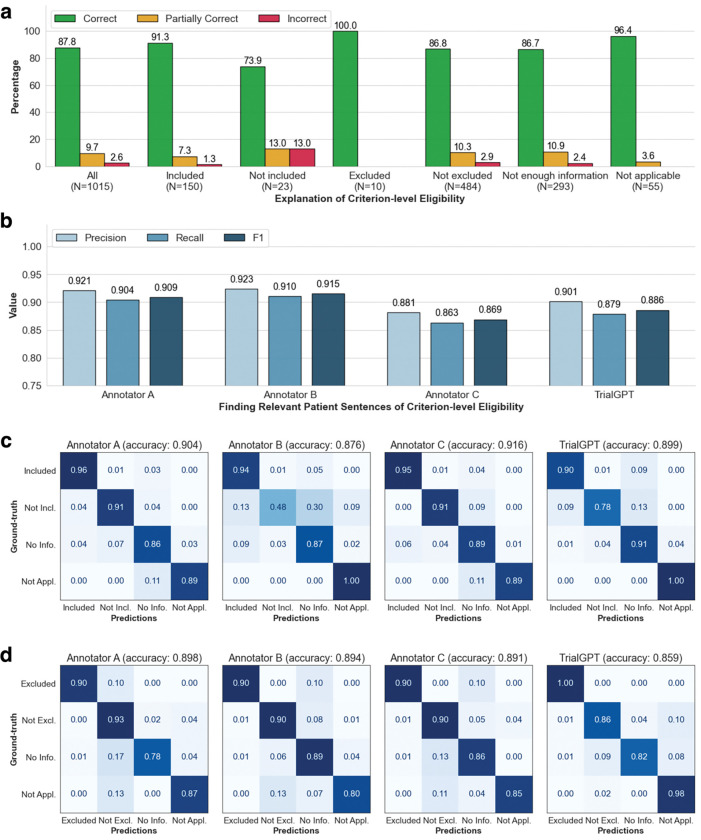
Manual evaluations of criterion-level predictions by TrialGPT. **(a)** The percentage of correct, partially correct, and incorrect relevance explanations generated by TrialGPT; **(b)** Evaluation results of the relevant sentences located by TrialGPT; **(c)** The confusion matrices of the eligibility for inclusion criteria predicted by human experts and TrialGPT; **(d)** The confusion matrices of the eligibility for exclusion criteria predicted by human experts and TrialGPT. Not Incl.: Not included. Not Excl.: Not excluded. No Info.: Not enough information. Not Appl.: Not applicable.

We show the percentage of “correct”, “partially correct” and “incorrect” TrialGPT explanations in Figure 2a. Overall, most explanations are “correct” (87.8%) by manual evaluations, while less than 10% of explanations are “partially correct” (9.66%), and only a small proportion are “incorrect” (2.56%). We also found that most of the incorrect explanations are for criteria labeled as “not included” and “not excluded”, which usually require implicit inference. TrialGPT exhibits much fewer mistakes when the criteria are explicitly “included” or “excluded”. These results suggest that TrialGPT can effectively explain how a patient is relevant to an eligibility criterion.

### Evaluating relevant sentence locations

We further compare the relevant sentences predicted by TrialGPT against the ground-truth expert annotations of relevant sentence locations. As shown in Figure 2b, the TrialGPT-predicted sentence locations are 90.1% correct (precision) and cover 87.9% of the ground-truth relevant sentence IDs (recall), leading to an F1 score of 88.6%. The performance of TrialGPT is close to that of human experts, ranging from 86.9% to 91.5%. This shows that TrialGPT can faithfully locate relevant sentences in patient notes, which further improves TrialGPT's explainability.

### Evaluating eligibility prediction

Finally, we evaluate the criterion-level eligibility labels predicted by TrialGPT and individual human annotators against the ground-truth annotations. Figures 2c and 2d show the confusion matrices for these predictions. For the inclusion criteria, TrialGPT reaches a prediction accuracy of 0.899 for all four labels, which is within the experts' accuracy range from 0.876 to 0.916. For the exclusion criteria, while the accuracy is high for criteria labeled with “excluded” (1.00) and “not applicable” (0.98), TrialGPT tends to confuse among “not excluded”, “no relevant information”, and “not applicable”. TrialGPT achieves an accuracy of 0.859 on the exclusion criteria. These results suggest that TrialGPT can accurately predict patient eligibility at the criterion level, with a performance close to that of human experts.

### TrialGPT mostly makes reasoning-related errors

We further inspected the 26 criterion-level predictions that are labeled as “Incorrect” by annotator consensus. Four types of errors have been identified: (E1) Incorrect reasoning, where TrialGPT predicts “not enough information” but the matching result can be implicitly inferred; (E2) Lack of medical knowledge, such as not knowing “A” is “B” or “A” is a type of “B”; (E3) Ambiguous label definitions, where the model confuses between two annotations, e.g., “not enough information” and “not applicable”; (E4) Other unclassified errors. Supplementary Table 1 shows the proportion and example of each error type made by TrialGPT. Most (30.7%) of the errors are due to incorrect reasoning, followed by ambiguous or redundant definitions of the eligibility classes (26.9%). Lack of medical knowledge contributes to about 15.4% of the total errors. These results suggest that improving the medical capabilities of the backbone LLM is an important future direction.

### Aggregated TrialGPT scores correlate with trial-level eligibility

**Figure 3.**
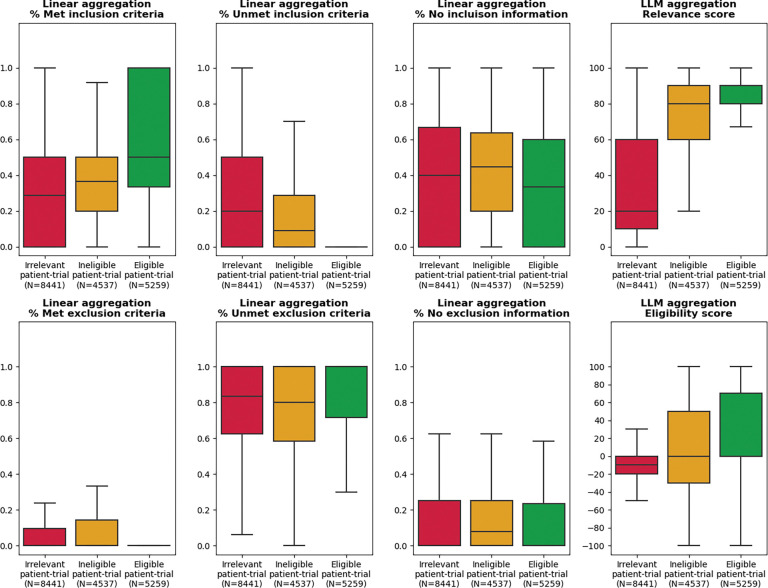
Correlation between differently aggregated TrialGPT scores and the ground-truth patient-trial eligibility labels. **(a)** The percentage of inclusion criteria predicted as “included” by TrialGPT; **(b)** The percentage of inclusion criteria predicted as “not included”; **(c)** The percentage of inclusion criteria predicted as “no relevant information”; **(d)** The percentage of exclusion criteria predicted as “excluded”; **(e)** The percentage of exclusion criteria predicted as “not excluded”; **(f)** The percentage of inclusion criteria predicted as “no relevant information”; **(g)** The LLM-aggregated relevance score; **(h)** The LLM-aggregated eligibility score. “**” denotes p < 0.01, “***” denotes p < 0.001, and “n.s.” denotes not significant (p > 0.05) by independent t-test.

TrialGPT has been shown to achieve high prediction accuracy at the criterion level. However, since one clinical trial typically has many inclusion and exclusion criteria, trial-level scores should be computed as a way to decide the extent to which a given patient is eligible or ineligible. In this section, we evaluate how criterion-level predictions of TrialGPT can be aggregated into trial-level scores (component shown in Figure 1b). For this, we analyze the correlations between patient-trial eligibility and eight trial-level scores, which are computed by two types of methods: linear aggregations and LLM aggregations. The results are presented as box plots in Figure 3.

### Linear aggregations

Six scores are computed by counting the percentages of the criterion-level eligibility predictions of TrialGPT. Their correlations with trial-level eligibility labels are shown in Figure 3a–f. Figure 3a shows the percentage of inclusion criteria predicted as “included” by TrialGPT. As expected, Figure 3a implies that the patients meet the highest percentages of inclusion criteria in eligible clinical trials and meet the lowest percentages of inclusion criteria in irrelevant clinical trials. The percentage of met inclusion criteria falls in between for relevant but ineligible trials. Figure 3b shows the percentage of inclusion criteria predicted as “not included”, which follows the reverse trends of the met inclusion criteria (Figure 3a). Noticeably, no inclusion criterion is classified by TrialGPT as “not included” in eligible patient-trial pairs, confirming the correctness of the model. Figure 3d shows the percentage of exclusion criteria predicted as “excluded”. Interestingly, patients meet more exclusion criteria in ineligible clinical trials than in irrelevant and eligible clinical trials, unlike other graphs that are either monotonically increasing or decreasing with regard to the irrelevant-ineligible-eligible order. This is a characteristic feature of patient-trial pairs that are explicitly excluded and can be exploited in patient-trial matching.

### LLM aggregations

We also use LLMs to further aggregate the criterion-level predictions of TrialGPT, resulting in two scores. The general relevance score (0~100) is shown in Figure 3g, where the irrelevant patient-trial pairs are much lower than the other two groups. Eligible and ineligible patient-trial groups have certain overlaps, but the former is still significantly higher than the latter. The eligibility score (−100~100) is shown in Figure 3h, where negative scores denote ineligible, positive scores denote eligible, and a score of 0 denotes neutral. Overall, the eligible patient-trial pairs have the highest scores, and the ineligible patient-trial pairs have the lowest scores.

In summary, criterion-level TrialGPT predictions can be aggregated into trial-level scores that are highly correlated with patient-trial eligibility. The results of linear aggregations demonstrate that eligible patient-trial pairs have the highest proportions of met inclusion criteria and unmet exclusion criteria, while ineligible patient-trial pairs have the highest proportions of met exclusion criteria. In addition, the LLM aggregations are also significantly correlated with the manual eligibility labels. These results suggest that the aggregated scores of TrialGPT can be potentially used to rank or exclude a list of candidate clinical trials for a given patient.

### TrialGPT can effectively rank and exclude candidate clinical trials

In this section, we evaluate TrialGPT on ranking a list of candidate clinical trials and excluding ineligible clinical trials for a given patient (component Figure 1c). Based on the correlation analysis, we design a suite of scoring methods to aggregate criterion-level predictions of TrialGPT to generate a trial-level score for ranking the candidate trials. Table 2 shows the Normalized Discounted Cumulative Gain at rank 10 (NDCG@10), Precision at rank 10 (P@10), and Area Under the Receiver Operating Characteristic curve (AUROC) of different methods in comparison to state-of-the-art models, which are described in Online Methods.

### Ranking candidate clinical trials

As shown in Table 2, TrialGPT outperforms all compared baselines, including dual-encoder, cross-encoder, and encoder-decoder models trained on different biomedical and clinical natural language inference (NLI)^30^ datasets. The best baseline for ranking clinical trials is the cross-encoder BioLinkBERT^31^ trained on MedNLI^32^, which achieves the NDCG@10 of 0.5558 and the P@10 of 0.4663. The most effective features of TrialGPT for ranking are the LLM-aggregated scores. They achieve NDCG@10 of 0.7339 (by Relevance) and P@10 of 0.5660 (by Eligibility), which are much higher than other aggregations. Combining both linear and LLM aggregations yields the highest-ranking performance, with the NDCG@10 of 0.8165 and the P@10 of 0.7328.

### Excluding ineligible clinical trials

Table 2 also shows the AUROC of excluding candidate trials, which is modeled as a binary classification task. The best baseline for excluding clinical trials is the dual-encoder SapBERT^33^ trained on MNLI^34^, SNLI^35^, SciNLI^36^, SciTail^37^, MedNLI^32^, and STSB^38^, achieving the AUROC of 0.5842. This result only shows marginal improvement over the random score baseline, indicating that the task of excluding ineligible trials presents significant challenges. Unlike in the task of ranking clinical trials, the percentage of inclusion criteria predicted as “not included” and the percentage of exclusion criteria predicted as “excluded” also achieve comparable AUROC individually. Again, TrialGPT outperforms all baselines, and the combination of the features achieves an AUROC of 0.7749.

These experimental results show that TrialGPT can effectively rank candidate clinical trials and exclude ineligible clinical trials, which could facilitate the trial-matching process.

### TrialGPT can reduce the screening time for patient-trial matching

**Figure 4.**
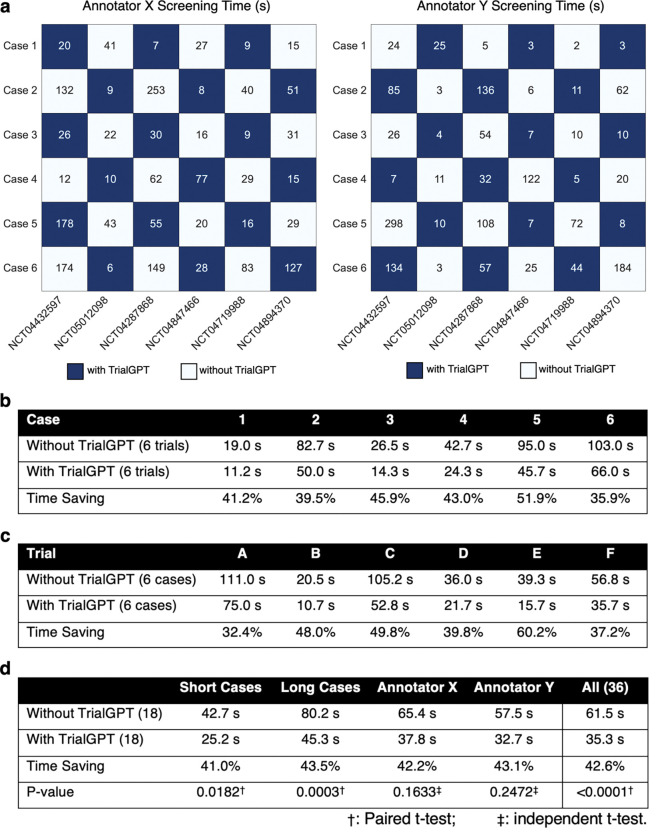
Results of the patient-trial matching user study. **(a)** Experimental design and actual screening times of each patient-trial pair by two expert annotators; **(b)** Comparison of screening time aggregated by different clinical trials; **(c)** Comparison of screening time aggregated by different patient cases; **(d)** Comparison of screening time aggregated by short cases, long cases, annotators, and all pairs. Numbers in parentheses denote the sample sizes in the corresponding group of the comparison. Within the annotator (e.g., Annotator X or Y), significant tests are conducted by independent t-test. Other significant tests are paired t-tests. Trial A, B, C, D, E, and F denote NCT04432597, NCT05012098, NCT04287868, NCT04847466, NCT04719988, and NCT04894370, respectively.

To evaluate whether TrialGPT can assist clinicians in performing the patient recruitment task, we set up a user evaluation that mimics patient-trial matching in real clinical settings. The evaluation is shown in Figure 4a. Here, we consider six oncology clinical trials. Among these trials, four are conducted at the National Cancer Institute and have a physician coauthor (C.F.) as principal or associate investigator, and the other two are highly related. The physician also created six semi-synthetic clinical vignettes (cases 1 to 6, available in the Supplementary Material) based on actual, anonymized patient referral requests. Cases 1–3 are short summaries of patients, while cases 4–6 are more extensive descriptions. For each of the patient-trial combinations, the matching task is to quickly screen the eligibility criteria to either reject the patient or include the patient for further investigation. Two medical doctors (Annotators X and Y) performed the tasks, where half of the patient-trial pairs have TrialGPT predictions and the other half do not have them. We also ensure that for each patient-trial pair, one annotator screened with TrialGPT, and another without. The accuracy of annotations with TrialGPT is higher than without (97.2% vs. 91.7%), although both are above 90% and their differences are not statistically significant. The comparison results for matching efficiency are shown in Figure 4. We observe a consistent trend of improved efficiency with TrialGPT: 35.9% to 51.9% less time is spent for different patients, and 32.4% to 60.2% less time is spent among different trials. In general, more time saving is observed for long cases (43.5%) than for short cases (41.0%). The overall time saving for all patient-trial pairs is about 42.6%, which can greatly improve the efficiency of a team evaluating patient referrals for clinical trials.

## Discussion

We present a systematic evaluation of LLMs for patient recruitment in clinical trials. For this, we propose TrialGPT, a new framework for patient-trial matching with LLMs. The technical novelty of TrialGPT is to utilize LLMs for two vital sub-tasks in clinical trial matching: (1) Criterion-level prediction: TrialGPT can match patient and clinical trials at criterion-level with explanations, where previous models suffer from the lack of annotated instances and cannot provide explanations; (2) Aggregation of criterion-level predictions: TrialGPT further utilizes LLMs to aggregate the criterion-level predictions to generate trial-level scores, which outperforms other linearly aggregated scores at ranking and excluding candidate clinical trials. The language understanding capability of LLMs is exploited in both tasks, and the language generation capability of LLMs lays the foundation for the explainability of the overall workflow.

For evaluation, we use three publicly available patient-trial matching datasets, where the patients are represented by a paragraph of free-text clinical summary. However, clinical trial matching sometimes requires the recruiters to check the patients’ information more comprehensively, which involves longitudinal clinical notes, lab values, and even imaging data. This requires the model to (1) attend to much longer contexts, (2) process structured data, and (3) process multi-modal inputs. These aspects have not been evaluated by this study but are worth exploring in future work. We also notice that the overall task formulation of the SIGIR and TREC datasets might be over-simplified, as many trial restrictions are not considered (geolocations, recruitment status, etc.), and the eligibility annotation is not strict at handling criteria with no relevant information.

Our work supports the position that the AI models for clinical trial matching should not be designed to replace human recruiters but to empower them, and experts should always be in the loop of medical AI deployments. Evaluation in real-life clinical trial matching scenarios should also focus more on efficiency improvement for human recruiters, instead of solely reporting the prediction performance. In this sense, the explanation capability of TrialGPT, or more generally LLMs, is particularly helpful. This is exemplified by our pilot user study showing that TrialGPT can significantly reduce 42.6% of the screening time on average.

While our experiments have shown promising results in patient-to-trial matching with TrialGPT, this study has two main limitations. First, TrialGPT relies on GPT-4 as the backbone model. Although GPT-4 is the most capable LLM, it is closed-source and can only be accessed via commercial API. Future studies should explore using and fine-tuning other open-source LLMs as alternatives. Second, our pilot user study is of limited scope in sample size. Nonetheless, it offers insights into the potential benefits of LLMs for assisting clinical trial matching and provides impetus to conduct larger-scale prospective evaluations with regard to the impact of LLM-assisted clinical workflows in future studies.

In summary, we present TrialGPT, a first-of-its-kind architecture that uses large language models to perform patient-trial matching. Our systematic evaluations show that TrialGPT can accurately predict criterion-level eligibility with faithful explanations. We also explore different pooling methods to aggregate the criterion-level predictions to trial-level scores that can be used to rank or exclude a list of candidate trials. The pilot user study has clearly demonstrated that TrialGPT significantly reduces the screening time needed for human experts. As such, we anticipate that large language models can be valuable in assisting the process of patient-trial matching.

## Online Methods

### Patient cohorts

We use three publicly available cohorts in this study: the SIGIR 2016 cohort, the TREC 2021 CT cohort, and the TREC 2022 CT cohort. Following the TREC CT tracks, we collect three eligibility labels for each patient-trial pair: (0) irrelevant, where the patient is “not relevant to the trial in any way”; (1) excluded, where the patient is explicitly excluded; and (2) eligible, where the patient is eligible to enroll in the trial. All three cohort annotations only use the patient note and eligibility criteria without considering the geolocations and recruitment status of the clinical trials.

### SIGIR 2016

The original cohort contains 60 patient case reports, but 1 report is removed since the report is about a group of patients (topic ID 201426, “A group of 14 humanitarian service workers…”). The patient notes are derived from the Clinical Decision Support (CDS) tracks in TREC 2014 and 2015, which are “medical case narratives created by expert topic developers that will serve as idealized representations of actual medical records”^43^. They typically describe the patienťs medical history, current symptoms, tests, eventual diagnosis, and treatments. Given a patient note, four medical assessors annotate each candidate clinical trial with three possible labels: (a) “would not refer this patient for this clinical trial”; (b) “would consider referring this patient to this clinical trial upon further investigation”; and (c) “highly likely to refer this patient for this clinical trial”. We consider the label a to be “irrelevant” and the label c to be “eligible”. Patient-trial pairs with the label b are not used in this work. The candidate clinical trials for annotation are derived from pooling various retrieval methods.

### TREC 2021/2022 CT

The TREC 2021 and 2022 CT tracks contain 75 and 50 patients, respectively. These patient notes are synthetic patient case descriptions, “such as what may be included as part of an admission note”. For each patient, they annotate three eligibility labels for the candidate clinical trial: irrelevant, excluded, and eligible. The candidate clinical trials are pooled from the submission systems of TREC participants.

### TrialGPT

TrialGPT is an architecture for patient-trial matching with large language models. It is composed of three modules: 1) a backbone LLM, 2) a criterion-level prediction module, and 3) a trial-level aggregation module. Here we denote a patient note as a list of P sentences $\left[s_{1},s_{2},\ldots ,s_{P}\right]$, a clinical trial as composed of the background information Β (containing the title, conditions, interventions, and the brief summary), a list of M inclusion criteria $\left[i_{1},i_{2},\ldots ,i_{M}\right]$, and a list of N exclusion criteria $\left[e_{1},e_{2},\ldots ,e_{N}\right]$.

### Backbone LLM

TrialGPT is LLM-agnostic, meaning it can be plugged into different backbone LLMs. In this study, we use the GPT-4 API (model index: gpt-4, 0613 version, 8k context length) through Microsoft Azure’s OpenAI services. We set the inference temperature to 0 for deterministic outputs.

### Criterion-level prediction

The objective of this module is to output a free-text relevance explanation *R*, a list of relevant sentence IDs *S*, and the eligibility prediction *E* for each criterion based on the input patient note. For an inclusion criterion,

*E* ∈ {included, not included, not enough information, not applicable},

while for an exclusion criterion,

*E* ∈ {excluded, not excluded, not enough information, not applicable}.

We use different label sets for inclusion and exclusion criteria because the latter are often ambiguous. For example, exclusion criteria of “Pregnancy,” “The patient should not be pregnant,” and “Pregnant patients will be excluded” serve the same purpose. Traditional entailment labels might not be suitable to distinguish the semantic differences, while our eligibility-oriented label sets provide an end-to-end solution.

We make two LLM inference calls for each patient-trial pair: one for all inclusion criteria, and another one for all exclusion criteria. Overall, the prompt includes the task description, the clinical trial background information B, and the inclusion $\left(\left[i_{1},i_{2},\ldots ,i_{M}\right]\right)$ and exclusion $\left(\left[e_{1},e_{2},\ldots ,e_{N}\right]\right)$ criteria. Motivated by chain-of-thought prompting^44^, we prompt the model to first generate the relevance explanation as grounding for future predictions of the relevant sentence IDs and the eligibility labels. In addition, we also prompt the model to generate criterion-level predictions in the JSON format, which can be easily parsed for aggregations. The TrialGPT prompts are shown in Supplementary Tables 2 and 3.

### Trial-level aggregation

After getting the criterion-level predictions, TrialGPT then aggregates such scores to generate a trial-level score that can be used for practical applications such as ranking and excluding clinical trials. Specifically, we denote the eligibility predictions of TrialGPT for the inclusion criteria and exclusion criteria as $\left[E\left(i_{1}\right),E\left(i_{2}\right),\ldots ,E\left(i_{M}\right)\right]$ and $\left[E\left(e_{1}\right),E\left(e_{2}\right),\ldots ,E\left(e_{N}\right)\right]$, respectively.

#### Linear aggregation:

six scores are simply derived based on the percentages of different eligibility predictions. While more sophisticated scoring methods can be used, we intentionally use these simple and linear aggregation strategies for better probing the capabilities of LLMs.

For a trial’s inclusion criteria:

$$\%\;\text{met}\;\text{inclusion}\;\text{criteria}=\frac{|\;\left\{E\left(i_{x}\right)\;=\;\text{included}\;|\;x\;=\;1\;,\ldots ,\;M\right\}\;|}{M^{\prime }}$$

$$\%\;\text{unmet}\;\text{inclusion}\;\text{criteria}\;=\frac{|\;\left\{E\left(i_{x}\right)\;=\;\text{not}\;\text{included}\;|\;x\;=\;1\;,2\;,\ldots ,\;M\right\}\;|}{M^{\prime }}$$

$$\%\;not\;enough\;information\;about\;inclusion\;criteria=\frac{|\;\left\{E\left(i_{x}\right)\;=\;\text{not}\;\text{enough}\;\text{information}\;|\;x\;=\;1\;,2\;,\ldots ,\;M\right\}|}{M^{\prime }}$$

$$M^{\prime }=M-|\left\{E\left(i_{x}\right)=\;\text{not}\;\text{applicable}\;|x=1\;,2\;,\ldots ,M\right\}|$$

For a trial’s exclusion criteria:

$$\%\;\text{met}\;\text{exclusion}\;\text{criteria}\;=\frac{|\left\{E\left(e_{y}\right)\;=\;\text{excluded}\;|\;y\;=\;1\;,2\;,\ldots ,\;N\right\}|}{N^{\prime }}$$

$$\%\;\text{unmet}\;\text{exclusion}\;\text{criteria}\;=\frac{|\;\left\{E\left(e_{y}\right)\;=\;\text{not}\;\text{excluded}\;|\;y\;=\;1\;,2\;,\ldots ,\;N\right\}\;|}{N^{\prime }}$$

$$\%\;not\;enough\;information\;about\;exclusion\;criteria=\frac{|\;\left\{E\left(e_{y}\right)\;=\;\text{not}\;\text{enough}\;\text{information}\;|\;y\;=\;1\;,2\;,\ldots ,\;N\right\}\;|}{N^{\prime }}$$

$$N^{\prime }=N-|\;\left\{E\left(e_{y}\right)=\;\text{not}\;\text{applicable}\;|x=1\;,2\;,\ldots ,N\right\}\;|$$

#### LLM aggregation:

In addition, TrialGPT also uses LLMs to aggregate the criterion-level predictions by the prompt shown in Supplementary Table 4. The generated scores are directly used as aggregation scores. Specifically, we consider two main features: general relevance and eligibility: The general relevance (R) indicates how relevant a patient is to clinical trial, while the eligibility score (S) denotes how eligible the patient is to the clinical trial. We restrict that:

0≤R≤100

where 0 indicates that the patient is irrelevant to the clinical trial and 100 suggests that the patient is exactly relevant to the clinical trial. We further restrict that:

−R≤S≤R

based on the assumptions that the absolute value of eligibility cannot be higher than relevance. We adopt self-consistency prompting^45^ with five calls and use the average scores.

#### Feature combination:

We further combine the linear and LLM aggregation features, generating the improved scores for ranking and excluding:

combination=I(%unmetinclusioncriteria>0)+I(%metexclusioncriteria>0)−%metinclusioncriteria−%LLMgeneralrelevance−%LLMeligibilityscore

where I is an indicator function:

$$I(\text{condition})=\left\{\begin{array}{ll} 1, & \text{if}\;\text{condition}\;\text{is}\;\text{True}\\ 0, & \text{if}\;\text{condition}\;\text{is}\;\text{False} \end{array}\right.$$

### Criterion-level expert evaluation

Three annotators (F.C., C.G., and C.F.) are provided with 1,015 pairs of patient-criterion predictions by TrialGPT sampled from our patient cohorts. For each patient-criterion pair, the annotators first evaluate the correctness of TrialGPT explanation by “Correct”, “Partially Correct” or “Incorrect”. If at least two annotators provide the same label, it will be used as the consensus. If annotators choose three different scores for a patient-criterion pair, it will be labeled as “Partially Correct”. The annotators then annotate the relevant sentence locations for the criterion, and the consensus is the union of all annotator-provided sentences. Finally, the annotator provides the annotation of eligibility, with the same candidate label set as that of TrialGPT. Similarly, if at least two annotators assign the same eligibility label, it will be used as the consensus. If all three annotators assign different eligibility labels, a second round of discussion will be scheduled until there is a consensus label.

### Compared methods

The core of TrialGPT lies in the prediction of patient-criterion eligibility. These predictions are then aggregated for the ranking and excluding tasks. As such, we compare TrialGPT to a variety of pre-trained language models that can predict patient-criterion eligibility. Since there are no existing patient-criterion eligibility annotations for training a supervised model, we consider transfer learning from the biomedical NLI datasets. Specifically, we use three categories of baselines: dual-encoder, cross-encoder, and encoder-decoder.

Dual-encoder models are also known as bi-encoder, where the patient note and the criterion are separately encoded by pre-trained transformers, and the eligibility is modeled as the similarity of the encoding vectors:

$$\text{score(ranking)}=\frac{\sum_{x=1}^{M}\;\;V(\text{patient}{)}^{T}V\left(i_{x}\right)}{M}-\frac{\sum_{y=1}^{N}\;\;V(\text{patient}{)}^{T}V\left(e_{y}\right)}{N}$$

$$\text{score(excluding)}=\frac{\sum_{y=1}^{N}\;\;V(\text{patient}{)}^{T}V\left(e_{y}\right)}{N}$$

and:

$$V(\text{patient})=Enc\left(\left[s_{1},s_{2},\ldots ,s_{P}\right]\right)\in R^{h}$$

$$V\left(i_{x}\right)=Enc\left(i_{x}\right)\in R^{h}$$

$$V\left(e_{y}\right)=Enc\left(e_{y}\right)\in R^{h}$$

where Enc denotes the pre-trained transformer encoder, and h is the dimension of the vector representations.

Cross-encoder models take both the patient note and the criterion as input, which enables cross-attention computations between the tokens in both texts. The eligibility prediction is modeled as a 3-way classification task based on the special [CLS] embedding of BERT^46^. We use label space mapping functions f that maps an NLI label to an eligibility label:

$$E\left(i_{x}\right)=f_{\text{inc}}\left(CrossEnc\left(\left[s_{1},s_{2},\ldots ,s_{P}\right],i_{x}\right)\right)$$

$$E\left(e_{y}\right)=f_{\text{exc}}\left(CrossEnc\left(\left[s_{1},s_{2},\ldots ,s_{P}\right],e_{y}\right)\right)$$

where

$$f_{\text{inc}}(l)=\left\{\begin{array}{c} \text{included,}\;\text{if}\;l=\text{entailment}\\ \text{not}\;\text{included,}\;\text{if}\;l=\text{contradiction}\\ \text{no}\;\text{relevant}\;\text{information,}\;\text{if}\;l=\text{neutral} \end{array}\right.$$

and

$$f_{\text{exc}}(l)=\left\{\begin{array}{c} \text{excluded,}\;\text{if}\;l=\text{entailment}\\ \text{not}\;\text{excluded,}\;\text{if}\;l=\text{contradiction}\\ \text{no}\;\text{relevant}\;\text{information,}\;\text{if}\;l=\text{neutral} \end{array}\right.$$

Then we compute the combination scores based on the criterion-level prediction, similar to the feature combination strategy used by TrialGPT:

$$combination\left(ranking\right)\;\;=\%\;met\;inclusion\;criteria\;-\%\;unmet\;inclusion\;criteria\;\;-\%\;met\;exclusion\;criteria\;+\%\;unmet\;exclusion\;criteria$$

$$combination\left(excluding\right)\;\;=I\left(\%\;unmet\;inclusion\;criteria\;>0\right)+I\left(\%\;met\;exclusion\;criteria\;>0\right)\;\;-\%\;met\;inclusion\;criteria$$

Encoder-decoder models also take both the patient note and the criterion as input to the encoder, but instead of outputting a classification prediction, they generate the predicted NLI labels, e.g., “entailment”, “contradiction”, or “neutral”. These NLI labels are then mapped to eligibility labels that will be aggregated into the combination scores by the same methods described above for cross-encoder models.

### Evaluation settings

We report the NDCG@10 and P@10 for ranking candidate clinical trials, and AUROC for excluding ineligible clinical trials.

For computing NDCG@10 and P@10, we denote the ranked list of clinical trials as $\left[c_{1},c_{2},\ldots ,c_{T}\right]$, where T is the number of considered candidates. Their relevance scores are denoted as $\left[r_{1},r_{2},\ldots ,r_{T}\right]$, which are converted following the settings of the TREC Clinical Trials tracks:

$$r_{i}=\left\{\begin{array}{c} 0,\;\text{if}\;E\left(c_{i}\right)=\;\text{irrelevant}\\ 1,\;\text{if}\;E\left(c_{i}\right)=\;\text{ineligible}\\ 2,\;\text{if}\;E\left(c_{i}\right)=\;\text{eligible} \end{array}\right.$$

NDCG@10 is a measurement ranking quality, which is computed by:

$$NDCG@k=\frac{DCG@k}{IDCG@l}$$

where

$$DCG@k=\sum_{x=1}^{k}\;\frac{r_{x}}{{log}_{2}(i\;+\;1)}$$

and

$$IDCG@k=\sum_{x=1}^{k}\;\frac{{r^{\prime }}_{x}}{{log}_{2}(i\;+\;1)}$$

where $\left[{r^{\prime }}_{1},{r^{\prime }}_{2},\ldots ,{r^{\prime }}_{T}\right]$ denotes the relevance of an ideal ranking.

P@10 is another metric for ranking quality, computed by:

$$P@k=\frac{\sum_{x=1}^{k}\;\;r_{x}}{max\;(ℛ)\;\times \;k}$$

where ℛ denotes the set of relevance labels.

We draw the receiver operating characteristic (ROC) curve and compute the area under the ROC curve (AUROC) values using the sklearn package in Python.

### Pilot user study

The pilot user study mimics a common task at the cancer center, where the trial organizer screens patient referral requests against the candidate clinical trials. The patient note varies from a short paragraph in the email to a long document sent via fax. We consider six clinical trials (NCT04432597, NCT05012098, NCT04287868, NCT04847466, NCT04719988, and NCT04894370) as the candidates, where the first four are managed by one physician co-author (C.F.) and the other two are highly related. The physician also created six clinical vignettes (3 short and 3 long) based on real patient encounters, with the personally identifiable information modified. The task objective is to screen whether the patient is definitely ineligible (“No”) or potentially eligible and should be included for further investigation (“Maybe”). Two MD annotators (Q.J. and E.X.) recorded the time needed to make the decision after familiarizing themselves with the patient note. Each annotator screens half of the patient-trial pairs with TrialGPT and another half without. We also ensure that each patient-trial pair is screened by one annotator with TrialGPT and another without. The evaluation setting is visualized in Figure 4a.

## Figures and Tables

**Table 1.** Baseline statistics of the three patient cohorts used in this work. We show the mean ± standard deviation for applicable variables.

Cohort	SIGIR 2016	TREC 2021 CT	TREC 2022 CT
**N**	59	75	50
**Age (year)**	38.3 ± 23.5	41.6 ± 19.4	35.3 ± 20.2
**Gender (male: female)**	29: 30	38: 37	28: 22
**Note length (words)**	88.1 ± 36.8	156.2 ± 45.4	109.9 ± 21.6
**Eligible trials / patient**	6.5 ± 6.2	40.8 ± 13.8	38.3 ± 15.7
**Excluded trials / patient**	0.0	40.0 ± 14.8	32.7 ± 18.6
**Irrelevant trials / patient**	39.9 ± 11.3	50.0	50.0

**Table 2.** Performance of different methods for ranking and excluding clinical trials. The Sign() function assigns suitable signs for the corresponding task, e.g., for “% Included”, it will be “+” for ranking and “-” for excluding clinical trials.

Application			Ranking clinical trials		Excluding clinical trials
Method / Metric			**NDCG@10**	**P@10**	**AUROC**
Random score			0.3859	0.3629	0.4954
SciFive^39^ (encoder-decoder)		Further trained on MedNLI^32^	0.4461	0.3527	0.5591
BioBERT^40^ (dual-encoder)		Further trained^41^ on MNLI^34^, SNLI^35^, SciNLI^36^, SciTail^37^, MedNLI^32^, and STSB^38^	0.4622	0.4236	0.5806
PubMedBERT^42^ (dual-encoder)			0.4824	0.4421	0.5804
SapBERT^33^ (dual-encoder)	0.4651		0.4424	0.5842
BioLinkBERT^31^ (cross-encoder)		Further trained on MedNLI^32^	0.5558	0.4663	0.5522
TrialGPT criterion predictions	Linear aggr.	Sign(% Included)	0.6332	0.5750	0.6524
		Sign(% Not included)	0.5329	0.4978	0.6562
		Sign(% Excluded)	0.4370	0.4148	0.6513
		Sign(% Not excluded)	0.4541	0.4311	0.6279
	LLM aggr.	Sign(Relevance)	0.7339	0.5524	0.6496
		Sign(Eligibility)	0.7065	0.5660	0.6377
	**Feature combination**		**0.8165**	**0.7328**	**0.7749**

## Data Availability

The TREC Clinical Trial 2021 and 2022 cohorts can be downloaded from http://www.trec-cds.org/2021.html and http://www.trec-cds.org/2022.html, respectively. The SIGIR cohort is publicly available at https://data.csiro.au/collection/csiro:17152. The clinical vignettes for the user study are available in the Supplementary Materials.
